# Acquisition of terrestrial life by human ancestors influenced by forest microclimate

**DOI:** 10.1038/s41598-017-05942-5

**Published:** 2017-07-18

**Authors:** Hiroyuki Takemoto

**Affiliations:** Primate Research Institute, Kyoto University, Inuyama, 484-8506 Japan

## Abstract

Bipedalism, terrestriality and open habitat were thought to be linked to each other in the course of human evolution. However, recent paleontological evidence has revealed that early hominins evolved in a wooded, humid environment. Did the evolutionary process from arboreal to terrestrial life actually require open habitat? Here I report the seasonal change in forest utilization height of West African chimpanzees (*Pan troglodytes*) and central African bonobos (*Pan paniscus*), and show that the difference in terrestriality between these two species was mainly caused by ambient temperature differences between the two study sites. The key factor was the vertical structure of the microclimate in forests and its seasonality. The results suggest the possibility that human terrestrial life began inside a forest rather than in the savannah. Increasing seasonality and prolongation of the dry months throughout the late Miocene epoch alone could have promoted terrestrial life of our human ancestors.

## Introduction

The common ancestor of the *Pan* and human lineages is thought to have been an arborealist^1^ and all early hominin fossils have been found in forest patches or environments close to forests^2–6^. Fossils of *Ardipithecus ramidus*, an early-stage human ancestor after divergence from the *Pan* clade 4.4 millions years ago (mya), prove that this species adopted conditions involving terrestrial upright bipedality, and foraging in trees^7^. This species thus demonstrates that the human lineage never experienced knuckle walking like that of modern chimpanzees or gorillas^8^. Modern ape locomotion studies have shown that bipedalism can happen in an arboreal context to enable reaching flexible branches^9–11^, although modern ape bipeds are not accompanied by anatomical specializations for upright bipedal locomotion^12^. The above evidence may indicate that bipedalism, terrestriality and open habitat are not necessarily linked; and a partially terrestrial lifestyle in early hominins started in the time period when forests were still the main habitat for humans. The beginning of terrestrial life, in other words, will be explained independently from the establishment of bipedalism or expanding into open habitat. However, there have been no reports about the ecological factors contributing to the transition from arboreal to terrestrial life inside forests during the course of human evolution. Why did our hominin ancestors descend from the trees to the forest floor?

*Ardipithecus* teeth reveal that this species was more omnivorous than present-day chimpanzees^13^, and they ate fruit, nuts, tubers and animal foods such as insects, including some terrestrial foods, but feeding outside of the forest was rare^14^. We can conceive firstly that they sought foods on the forest floor or outside of the forest when food availability in the tree crown was scarce. In studies of present-day chimpanzees, however, the vertical distribution of the food supply cannot fully explain the seasonal change in terrestriality. Ground use in Taï chimpanzees, in West Africa, varied month to month and nut cracking behaviour on the ground was mentioned as a reason for this only for females in a particular month^15^. In Gombe, East Africa, though chimpanzees shift their feeding place to the ground in the dry season for eating shrub fruits or fallen fruits, they rest in trees in the wet season to avoid being affected by ground moisture^16^.

Feeding and resting places are almost independent in the Bossou forest, West Africa^17^. Terrestriality increases in the dry season not in order for chimpanzees to seek terrestrial foods, but rather to reduce the energy loss caused by the vertical structure of the microclimate. In general, the higher one goes up in the canopy, the more the air temperature rises and the more the relative humidity decreases compared to the ground surface during the daytime in a tropical forest^18^. Seasonal differences in the effective temperature appear to be narrow because animals move to higher places above the ground during the cool season and move nearer to ground level during the hot season. Diurnal analysis of ground usage in chimpanzees in a particular season clearly demonstrated that climatic variables affected their terrestriality in the Budongo forest, East Africa^19^.

The above four chimpanzee study sites, located in the peripheral zone of the African rain forest, have a seasonal climate with a significant dry season showing higher daytime temperature than the wet season^18^. Comparison of high and low seasonality habitats is needed to investigate the impact of temperature on *Pan* species’ terrestriality. This information will clarify how climate change historically influenced the early human lineage after an age of high seasonality arrived. Typical tropical rain forests, showing little seasonality, occupy the middle of central Africa, namely the Congo Basin, where bonobos live.

The Wamba area located in the core zone of the African tropical rain forest has a tropical wet or super-wet climate with 2,500 mm or more annual precipitation^20^ and no distinct dry months, though there are occasional short dry periods in January. Deciduous trees are rare in the primary forest. In contrast, annual rainfall at Bossou is around 2,200 mm and there are clear dry months from November to February owing to the proximity of the area to the northern edge of the tropical rain forest zone^21^. The climate there is defined as a tropical wet seasonal climate, and the forest contains a high proportion of deciduous trees^22^.

I compared space utilization in the wettest and driest months of Bossou chimpanzees and Wamba bonobos, using tree fruit availability (FAI) and forest microclimate as environmental factors. The activity budgets of six individuals at Wamba and four individuals at Bossou were recorded by following one individual for an entire day for as long as possible. Behaviour was categorized as arboreal feeding (AF); arboreal resting (AR); arboreal moving (AM); terrestrial feeding (TF); terrestrial resting (TR); and terrestrial moving (TM). Space utilization was evaluated as daily time spent on the ground (terrestriality: TF + TR + TM) and utilization height was recorded every ten minutes. The utilization height of an individual was expressed relative to the canopy top (relative height). Utilization heights for the focal animal were recorded instantaneously every 10 minutes by triangulation using a laser rangefinder (OptiLogic 400LH) and daily averages were calculated from this data.

**Figure 1:**
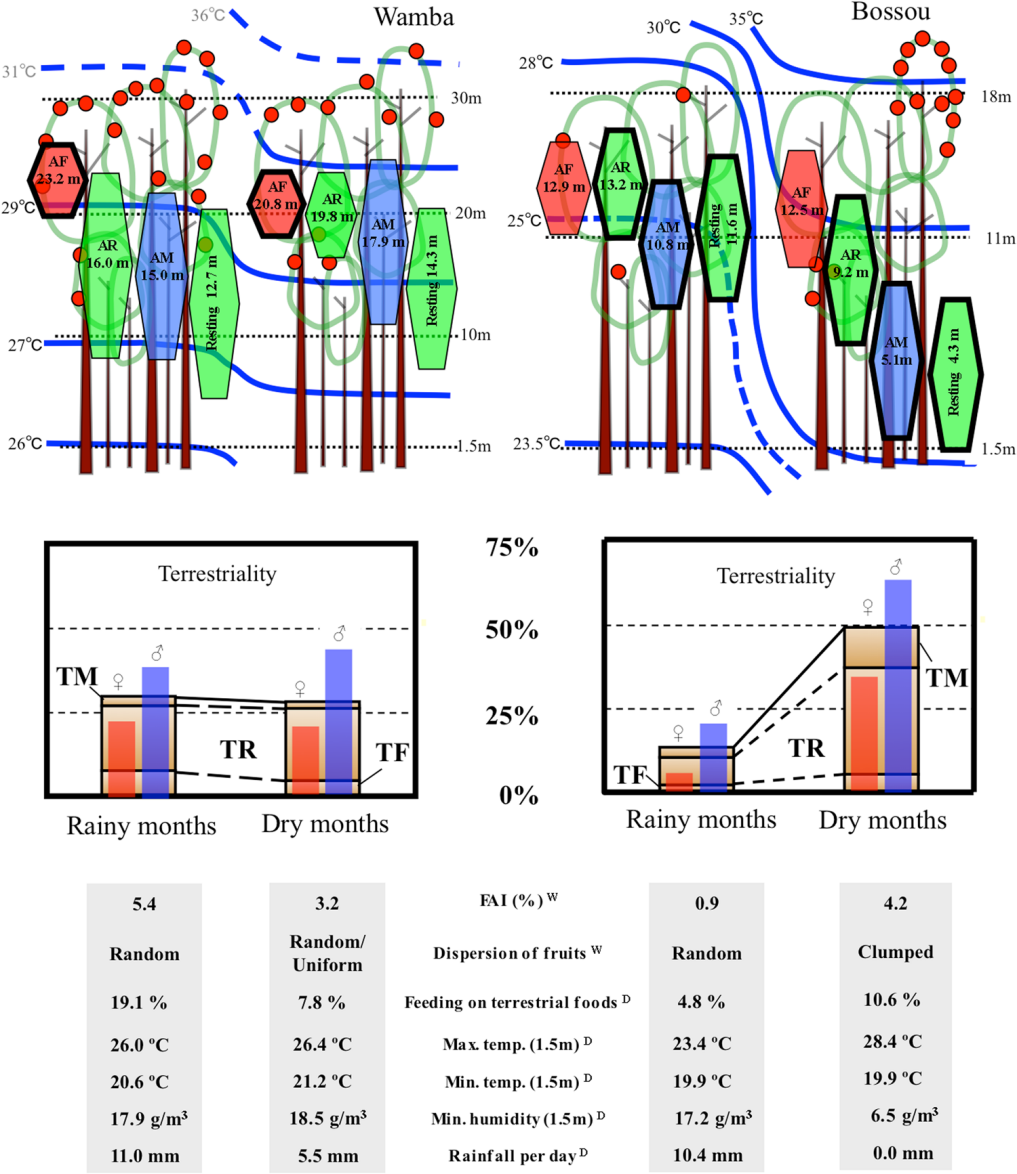
Scheme of the seasonal relationship between forest environments and utilization height or terrestriality in chimpanzees and bonobos. Upper: Seasonal changes in fruit availability and estimated air temperature contour lines. Broken lines mean alternative values were used when actual measurements were lacking (see Methods). Utilization heights were represented by height relative to the canopy where the forest microclimate was measured. Each vertical length of behaviour reflects the standard deviation. Behaviours surrounded by thick lines showed significant seasonal differences in each site. Middle: Time spent for terrestrial behaviour (%). Lower: Averages of environmental factors in each research period. AF: Arboreal feeding, AR: Arboreal resting, AM: Arboreal moving, Resting: Arboreal and terrestrial resting (AR + TR), D: Average of daily values, W: Every two-weeks’ values.

Not only air temperature but also other climatic variables, such as humidity or rainfall, may affect terrestriality. Clumped spatial patterns of fruit bearing trees may lengthen travel distance or time for moving between food patches. As a result, terrestriality may be strengthened. Some specific terrestrial foods for chimpanzees too, such as fallen nuts for cracking^15^, act as a motivating factor to move to the ground. The averages of these factors in each research period are shown in Fig. 1. Feeding time on terrestrial herbaceous vegetation (THV), ant dipping (a tool use behaviour for harvesting driver ants), and oil palm nut cracking were chosen as the effect of terrestrial foods for Bossou chimpanzees. Likewise, feeding time on THV and a species of subterranean mushrooms (local name, “Simbokilo”, which bonobos sometimes spend time foraging on in the swamp forest floor; ref. 23) were used as the terrestrial food effect for Wamba bonobos.

## Results

A clear trend in the vertical structure of air temperature was observed in both forests and in both research periods (Fig. 1). The canopy had daytime temperature about 5 °C~7 °C higher than that near the ground. The Bossou forest showed a greater seasonal difference in maximum temperature (>5 °C at same height) than the Wamba forest (<1 °C). The Bossou forest had a greater seasonal difference not only in air temperature but also in humidity, precipitation and fruit supply compared to the Wamba forest (Fig. 1). Dry months in Bossou showed high fruit availability and clumped distribution in fruiting trees, as well as a hotter and more arid microclimate. In contrast, the Wamba forest had little seasonal fluctuation in food supply and climate environment during the research periods.

The average utilization heights of all activity in Wamba bonobos did not differ between the two research periods: The values were 0.46 ± 0.25 S.D. in rainy months and 0.51 ± 0.17 in dry months (two-tailed Wilcoxon rank sum test, n_WR_ = 30, n_WD_ = 22, W = 381, p = 0.35) (hereafter, n_W_ = sample size for Wamba, n_WR_ = Wamba rainy months, n_WD_ = Wamba dry months, n_B_ = Bossou, n_BR_ = Bossou rainy months, n_BD_ = Bossou dry months). Similarly, there were no differences in AR (rainy months 0.53 ± 0.26, n_WR_ = 30, dry months 0.66 ± 0.13, n_WD_ = 21, W = 396, p = 0.12), AM (rainy 0.50 ± 0.25, n_WR_ = 18, dry 0.60 ± 0.23, n_WD_ = 18, W = 132, p = 0.33) or Resting (AR + TR) height (rainy 0.50 ± 0.25, n_WR_ = 30, dry 0.60 ± 0.23, n_WD_ = 22, W = 375, p = 0.41) (Fig. 1). There was a slight difference in AF height: It was higher when fruit was abundant (rainy 0.77 ± 0.11, n_WR_ = 28, dry 0.69 ± 0.10, n_WD_ = 21, W = 175, p = 0.0157). Utilization heights in Bossou chimpanzees were quite different from those in Wamba bonobos. The all-activity utilization height was higher in rainy months (0.65 ± 0.18) than in dry months (0.31 ± 0.18) (n_BR_ = 16, n_BD_ = 19, W = 34, p < 0.0001). There was no difference in AF height, but there was a significant difference in AR height (rainy 0.73 ± 0.14, n_BR_ = 16, dry 0.51 ± 0.23, n_BD_ = 19, W = 57, p = 0.0037), in AM height (rainy 0.60 ± 0.18, n_BR_ = 16, dry 0.29 ± 0.21, n_BD_ = 19, W = 15, p < 0.001) and in Resting height (rainy 0.65 ± 0.21, n_BR_ = 16, Dry 0.24 ± 0.23, n_BD_ = 19, W = 27, p < 0.0001).

Seasonal difference in terrestriality showed the same trends as utilization heights (Fig. 1). Terrestriality in Wamba was constant (rainy: 29.0 ± 22.7%, n_WR_ = 30, dry: 28.0 ± 23.2%, n_WD_ = 22, W = 325.5, p = 0.94), whereas Bossou chimpanzees used the ground for a much greater fraction of the time in dry months (50.1 ± 23.5%) than rainy months (13.5 ± 14.3%) (n_BR_ = 16, n_BD_ = 19, W = 278, p < 0.0001). All terrestrial behaviours (TF, TR and TM) of Bossou chimpanzees showed significantly larger time budgets in dry months than rainy months. On the other hand, those of Wamba bonobos showed no seasonal differences (Table 1). Bossou chimpanzees spent more time on the ground than did Wamba bonobos in dry months (n_BD_ = 19, n_WD_ = 22, w = 312, p = 0.0064), whereas less time was spent on the ground in rainy months (n_BR_ = 16, n_WR_ = 30, w = 139, p = 0.0204).

**Table 1:** Averages in activity budgets (% ± S.D.) stratified by research site and season.

Behaviour	c	Wamba				a	Bossou				d
		Rainy months (n = 30)	b	Dry months (n = 22)	Averages (n = 52)		Averages (n = 35)	Rainy months (n = 16)	b	Dry months (n = 19)	
AF	ns	17.71 ± 10.23	*	28.58 ± 14.94	22.31 ± 13.49	ns	23.67 ± 13.01	22.86 ± 15.36	ns	24.36 ± 11.05	ns
AR	ns	47.98 ± 23.06	ns	40.28 ± 25.56	44.72 ± 24.21	ns	39.52 ± 27.34	59.93 ± 22.76	***	22.33 ± 17.25	*
AM	ns	5.27 ± 3.21	*	3.15 ± 3.28	4.37 ± 3.38	ns	3.44 ± 2.30	3.68 ± 2.69	ns	3.23 ± 1.97	ns
TF	*	7.78 ± 10.15	ns	4.53 ± 7.03	6.30 ± 9.02	ns	4.06 ± 4.07	2.19 ± 2.98	**	5.64 ± 4.27	ns
TR	*	18.57 ± 16.88	ns	21.51 ± 20.15	20.25 ± 18.18	ns	21.86 ± 21.96	8.20 ± 10.83	***	33.37 ± 22.52	ns
TM	ns	2.12 ± 2.08	ns	1.95 ± 3.46	2.04 ± 2.72	***	7.45 ± 5.80	3.14 ± 2.93	***	11.08 ± 5.09	***

^a^p values by two-tailed Wilcoxon rank sum test between research sites. ^b^p values between two seasons within a research site. ^c^p values for rainy months between research sites. ^d^p values for dry months between research sites. *p < 0.05, **p < 0.01 ***p < 0.001.

**Figure2:**
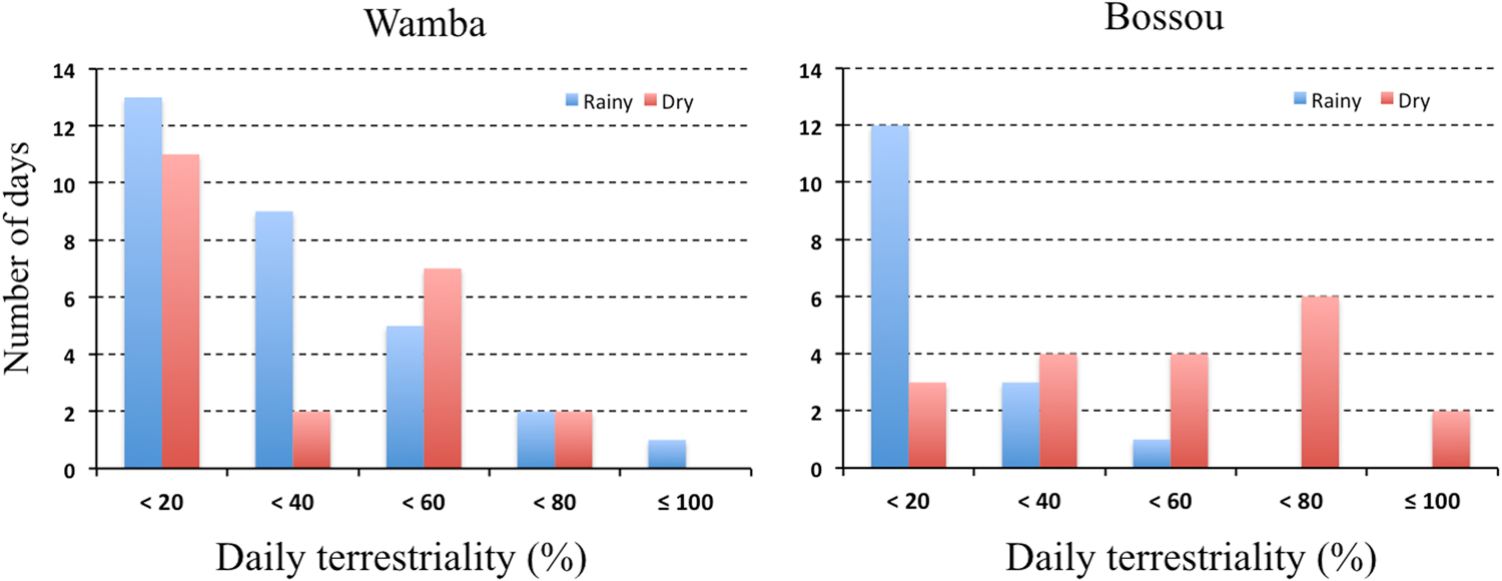
Seasonal diffence in the frequency of daily terrestriality (%). Blue bars: rainy months. Red bers: dry months.

Both bonobos and chimpanzees were observed on the ground in rainy months as well as dry months. Frequencies of daily terrestriality, however, varied between the research sites and seasons (Fig. 2). In Wamba, focal animals spent less than 20% of the time on the ground in a day on most days in both rainy and dry months. There was no seasonal difference (Fisher’s exact test, χ^2^ = 4.84, p = 0.304). In rainy months, Bossou chimpanzees spent less time (<20%) on the ground most days, and there were no days in which more than 60% of the time was spent engaged in terrestriality. They did not descend to the ground at all on two of the 16 observation days in the rainy months, whereas the least amount of daily terrestriality was 11.6% of 19 days observation in dry months. The peak frequency shifted from 0–20% in rainy months to 60–80% daily terrestriality in dry months (χ^2^ = 15.20, p = 0.00134).

**Figure3:**
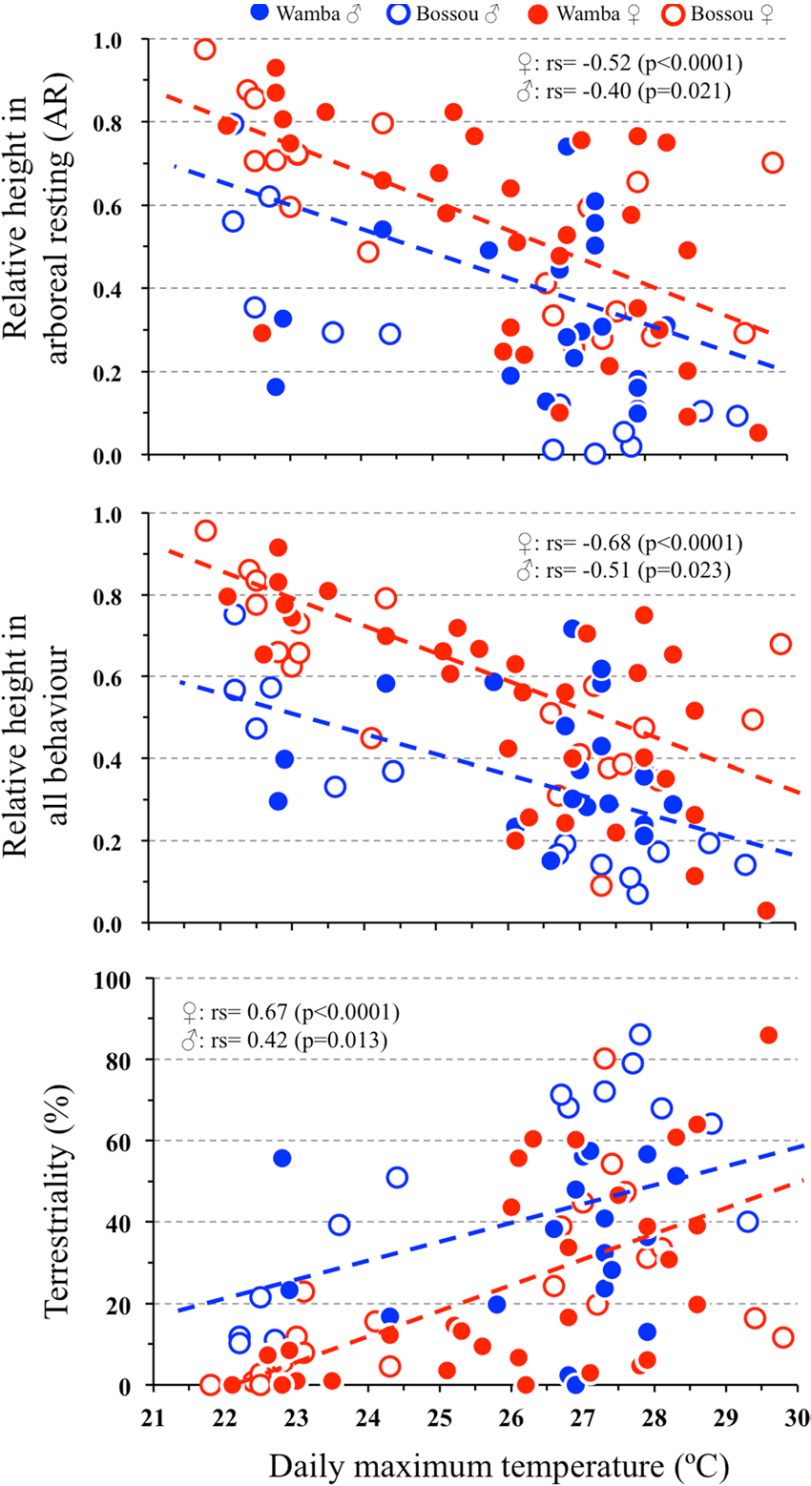
Correlation between utilization height (upper, middle) or terrestriality (lower) and daily maximum ambient temperature according to sex.

MCMCglmm using R was performed based on daily data to estimate which factor most strongly affected terrestriality (Table 2). Ambient temperature fluctuation inside the forest was the major factor affecting space utilization in both populations, except for sexual differences. The correlation between forest temperature and indices of space utilization is shown in Fig. 3. As maximum daytime temperature increased, *Pan* species’ utilization height gradually decreased and terrestriality increased. On hotter days, chimpanzees and bonobos spent more time on the ground and they used lower strata in the forests in each activity. There was no clear influence of other factors (vertical and horizontal distribution of food, rain or humidity, species or research site difference) on terrestriality. None of them showed the lowest pMCMC value in the single factor analyses or significant coefficients (post mean) in full factor models (Table 2).

**Table 2:** Results of MCMCglmm for terrestriality and utilization heights.

	Response variable	Full factor				Single factor					
		Terrestriality		Height (All behaviour)		Height (AF)		Height (AR)		Height (Resting)	
		DIC: 827.0		DIC: 820.3		DIC: 813.6		DIC: 807.6		DIC: 834.8	
	Factor	post mean	pMCMC	post mean	pMCMC	post mean	pMCMC	post mean	pMCMC	post mean	pMCMC
Foods	Fruit availability	−0.32	0.34	−0.03	0.92						
	Fruiting tree distribution (clumped)	1.34	0.35	−0.44	0.76						
	Terrestrial foods	0.49	0.58	−0.01	0.41						
Meteorology	**Max. Temp**.	**0.27**	**0.001**	−**0.36**	**< 0.001**			−**1.10**	**0.002**	−**0.43**	**< 0.001**
	Min.Temp.	0.14	0.47	0.07	0.71						
	Min.Humid.g/m^3^	−0.07	0.27	0.02	0.74						
	Daily Rainfall	−0.01	0.35	0.01	0.29						
	**Sex (♂)**	**1.08**	**0.001**	−**1.44**	**<0.001**	−**1.10**	**0.003**				
	Site (Wamba)	1.53	0.39	−0.19	0.91						

Full factor models were chosen for the analysis of “Terrestriality” and “Height of all behaviour”. Other three analysis for utilization heights were shown as single factor models.

Males showed greater terrestriality than females (male: 40.8 ± 23.2%, n = 34, female: 23.5 ± 23.1%, n = 52, w = 503, p = 0.0008; Fig. 1) and females used higher strata in forests (overall height; males: 0.31 ± 0.21, females: 0.53 ± 0.24, w = 1340, p < 0.0001) in both research sites. However, the trends of the response to ambient temperature were parallel in both sexes (Fig. 3). Males, having bigger body size than females, probably had difficulty to eat foods in the top branches of trees, as shown by the height of AF (Table 2). In addition, some socio-ecological factors, such as predation pressure might have caused this sex difference.

## Discussion

The factor “research site” contains various random effects between the two sites other than the factors listed in Table 2, such as species difference, topography, solar radiation, forest structure, soil water content and earth temperature. For example, the Wamba rain forest has a better developed stratum structure than the Bossou semi-deciduous forest, but the utilization heights of *Pan* species showed no apparent difference between both forests when the canopy heights was regulated in the same height (i.e. relative height analysis). Thus, these factors had little effect on the space utilization of these *Pan* species (Table 2). From the viewpoint of thermoregulation, there is no reason to consider that bonobos act differently from chimpanzees in response to ambient temperature. The lack of a large seasonal difference in air temperature in the Wamba forest might be the reason for the lack of a seasonal difference in terrestriality or resting height for bonobos (Fig. 1).

For the purpose of thermoregulation, decreasing utilization height and increasing terrestriality in *Pan* species are thought of as related phenomena. If terrestrial behaviour is influenced by some specific factors such as disliking wet ground^16^ or risk of infection by some parasites^24^, utilization height in trees need not have a correlation with ambient temperature. The thermoneutral zone of the ambient temperature for primates usually has a range of less than 5 °C, although the range differs depending on the species. The upper critical temperatures for anthropoids range from 25 °C to 34 °C and the lower critical temperatures range from 24 °C to 28 °C^17^. The thermoneutral zone for naked humans is reported to be from 25–27 °C to 28–31 °C^25^. It appears that a seasonal difference of 11.5 °C in the average maximum temperature (daily values ranged from 21.8 °C at 1.5 m height in wet months to 37.9 °C at the canopy in dry months) in the Bossou forest, exceeds physiological adaptations such as acclimatization. The seasonal shift in utilization heights might help to narrow down the range of acclimatization and reduce thermoregulation costs. The effective temperature range is made wider by solar radiation or rain. It is natural that endotherms would move to more comfortable places according to ambient temperature fluctuation.

Bonobos and chimpanzees tended to rest on the ground during midday in both research periods. This may be caused by diurnal changes in thermal conditions, which showed the maximum air temperature in the early afternoon. Daily time spent on the ground, however, was shorter in rainy months than in dry months by Bossou chimpanzees (Fig. 2). For example, since Bossou chimpanzees often fed up in the trees and rested on the ground in dry months, AM was observed mostly when they were climbing up or descending from a tree, that yielded a lower AM height in dry months (Fig. 1). On the other hand, they rested on the ground in a low frequency and rested in trees even at midday in rainy months. Therefore, AM height was not significantly different from AR height in the rainy season at Bossou (Fig. 1). Thus, diurnal changes in utilization height itself shifted seasonally and that generated greater time spent on the ground in dry months.

Both bonobos and chimpanzees have acquired a semi-terrestrial life style. Therefore, Wamba bonobos showed a certain degree of terrestriality in a certain stable thermal environment, helped by acclimatization or adaptation. *A*. *ramidus* is thought to have evolved from an arborealist, who may have adopted a low seasonal habitat spending little time on the ground. The appearance of higher daytime temperature months and a prolongation of them might have been enough for these arboreal ancestors to start a semi-terrestrial life, even if they spent much of the time in trees the rest of the year. In addition, a dry period of more than four months would result in the reduction of continuous forest areas. The extending of the dry months and forest area reduction combined probably further promoted terrestriality in hominins.

Most hypotheses about human evolution have considered climate change in the late Miocene, namely, a change to being cooler, drier with a higher degree of seasonality, than in the previous epoch, as a factor affecting habitat change from forest to savannah or savannah woodland^26^. Climate fluctuation, however, has been indicated to be one of the environmental drivers for human evolution^26, 27^. The present study showed a specific function of a strongly seasonal climate on the terrestriality of *Pan* species, and pointed out the possibility that the main factor behind habitat change, which led to human terrestrial life, was climate change itself.

Wetter and drier periods alternated under the influence of a monsoonal climate associated with the uplift of Tibet, and dry periods would have emerged at the peripheral zones of rain forests, such as in eastern North Africa^28^. At 9.9 to 9.3 mya in Africa, increasing C_4_ plants resulted from lower annual rainfall and a more pronounced dry season^29^. Climate seasonality became more pronounced toward the Pliocene^30^. This global aridification caused intermittent reductions of forest area through the middle to late Miocene. The peripheral area of the forest zone in the late Miocene should have had a seasonal climate, like that at Bossou today. The ancestor of hominins could not escape the effects of climate change in the forest, which was then their main habitat. They acquired a partial terrestrial lifestyle before the reduction of forest area. This may have served as a pre-adaptation for life on the woodland to savannah.

*A*. *ramidus* lived in patched semi-deciduous forest, occupied by palm trees^2^. That is a very similar environment to the forest of Bossou, which is a small semi-deciduous forest dominated by Moraceae species, including Ulmaceae such as *Celtis* spp, and is surrounded by savannah dotted with palm trees (*Elaeis guineensis*)^22^. It is thus possible to think that the ancestor of hominins acquired its terrestrial life habit in a strongly seasonal, isolated forest something not unlike the Bossou forest.

## Methods

The study was approved by the Field Research Committee of the Primate Research Insitute, Kyoto University. Data collection was approved by the Centre de Recherche en Ecologie et Forestrie (CREF) of Democratic Republic du Congo, Direction Nationale de la Recherche Scientifique et Technique (DNRST) and the Institut de Recherche Environnementale de Bossou (IREB), Republic du Guineé. All data including behavioural observation, phenology and meteorology were non-invasively collected.

The research was non-invasive and all methods were performed in accordance with the Association for the Study of Animal Behaviour guidelines. The wettest months for Wamba (N0′ 11″, E22′38″) were September to November 2008 and August to September 2007 for Bossou (N7′ 38″, W8′ 30). The driest months for Wamba were December to February 2005–2006 and 2007 and January to February 2008 for Bossou. The study sites of Wamba, in the Democratic Républic du Congo (representative rain forest habitat of bonobo in central Africa), and Bossou in the Républic du Guineé (representative seasonal forest chimpanzee habitat in west Africa), were chosen to compare utilization height and terrestriality. Data for Wamba dry months were combined from two different years. Group size was 24 for Wamba bonobos (E1 group) and 13 for Bossou chimpanzees at the beginning of this study.

The following methods were used at both sites, except for the analysis of the distribution of fruiting trees. The activity budgets of six individuals (three adult males and three adult females) for Wamba and four individuals (two adult males and two adult females) for Bossou were recorded by focal animal sampling and continuous recording methods, following one chimpanzee for an entire day or as long as possible to avoid introducing observation bias associated with grouping patterns, social behaviour, or visibility. Table 3 shows the total observation time for each focal animal. Utilization heights for the focal animal were recorded instantaneously every 10 minutes by triangulation using a laser rangefinder (OptiLogic 400LH) and the daily averages were calculated. Utilization height was always expressed as values of 0–1 (relative height to the canopy), wherever the study subject was found in the forests.

**Table 3:** Observation days and time for each focal animal.

Animal		Wet months		Dry months		Total	
		Days	Time	Days	Time	Days	Time
Wamba							
NB	♂	4	15:46:12	1	3:46:31	5	19:32:43
ND	♂	5	25:15:05	3	15:47:32	8	41:02:37
TW	♂	—	—	7	28:46:55	7	28:46:55
Jk	♀	6	19:16:03	—	—	6	19:16:03
No	♀	7	35:45:21	8	41:19:37	15	77:04:58
Yk	♀	8	38:04:22	3	7:34:10	11	46:38:32
Total		30	134:07:03	22	97:14:45	52	232:21:48
Bossou							
YL	♂	2	8:17:38	4	26:12:47	6	34:30:25
FF	♂	4	27:30:52	4	26:20:03	8	53:50:55
Jr	♀	6	34:49:13	7	40:37:48	13	75:27:01
Vl	♀	4	18:12:39	4	31:04:05	8	49:16:44
Total		16	88:50:22	19	124:14:43	35	213:05:05

Thermo-hygrometers (CHINO HN-CN, precision: ±0.5 °C, ±0.2%) with shade to protect against solar radiation and rain were set in selected trees at 1.5 m to near the tree crown in mature secondary forest at Bossou, and in primary forest at Wamba to examine seasonal differences in the vertical structure of the forest microclimate. Air temperature and absolute humidity were automatically recorded every 10 minutes and the daily maximum and minimum values were determined. At Bossou, because the thermo-hygrometers placed at 11 m height malfunctioned in the rainy months, so the temperature in the primary forest at the same height was used as a representative value in the figures. Because the air temperature at the canopy could not be measured in the Wamba forest, the temperature at a forest gap was substituted for the canopy temperature in the figures. The daily average wind velocity, measured using a Kestrel 4000 at 1.5 m and 10 m inside forests, might be negligible. Most days showed an average wind velocity of 0.0 m/s and maximum wind velocity exceeded 2 m/s on only two days at 10 m height in dry months at Wamba (3.3 m/s, 4.3 m/s).

I estimated tree fruit availability every two weeks in line transects established in the home range of each *Pan* species. The number of target trees more than 5 cm in DBH, regardless of life form such as liana or low tree, was 749 at Bossou and 104 at Wamba. Fruit abundance for each tree was ranked on a relative scale from 0–3, and FAI (fruit availability index (%)) was calculated, with the FAI when all trees were fully fruited (scale of 3) taken as 100%, as follows

1 $${\rm{FAI}}=[\sum ({\rm{Pi}}\times {\rm{Fi}})/\sum ({\rm{Pi}}\times 3)]\times 100$$

where Pi is the basal area of tree i (cm^2^), and Fi is the fruiting score of tree i (0–3).

The distribution of fruiting trees was tested using Morisita’s Index of dispersion as a unit 50 m in length and randomness was estimated.

2 $${{\rm{\chi }}}^{2}={{\rm{I}}}_{{\rm{\delta }}}(\sum {\rm{x}}-1)+{\rm{n}}-\sum {\rm{x}}\quad \quad ({\rm{d}}{\rm{.f}}{\rm{.}}={\rm{n}}-1)$$

where χ^2^ is the test statistic for Morisita’s index of dispersion (chi-square distribution), n is sample size, I_δ_ is Morisita’s index of dispersion, ∑x = sum of the fruiting trees. In Wamba, the fallen fruit census data of the Wamba research team, which ran along the same trail as the FAI transects, was used for calculating Morisita’s Index. The sample sizes were 41 (2,050 m) at Bossou and 56 (2,800 m) at Wamba.

MCMCglmm (MCMC generalized linear mixed models) in R package was performed to estimate factors affecting utilization height by *Pan* species. Family = “ordinal”, nitt = 1,000,000, thin = 100, burnin = 100,000, were set for the analysis.
